# Comparison of Modified Latarjet Procedure With Bone Graft Versus Standard Latarjet Procedure: Evaluating Outcomes and Stability

**DOI:** 10.7759/cureus.67908

**Published:** 2024-08-27

**Authors:** Vinod Nair, Mohammed Talha Muneer, Sahil Chowdhary

**Affiliations:** 1 Orthopedics, Dr. D.Y. Patil Medical College, Hospital and Research Centre, Pune, IND

**Keywords:** coracoid process, iliac crest, bone transplantation, latarjet procedure, shoulder instability

## Abstract

Background: The Latarjet procedure is a well-established technique for managing repeated anterior shoulder dislocation accompanied by massive glenoid bone loss. Aim of this article was to assess outcomes among modified Latarjet procedure using allograft from Iliac bone and a standard Latarjet procedure using the coracoid process.

Methods: Six patients with recurrent anterior shoulder instability and significant glenoid bone loss were retrospectively analyzed. Three patients underwent the modified Latarjet procedure with iliac crest bone graft (Group A), and three underwent the standard Latarjet procedure (Group B). Outcomes were assessed at the 12-month follow-up, including shoulder stability, functional scores (Constant, the American Shoulder and Elbow Surgeons (ASES) score, and the Western Ontario Shoulder Instability Index (WOSI)), range of motion, complications, and return to sports.

Results: Both groups showed improvements in stability and functional scores, with no recurrent instability reported. Group A demonstrated slightly higher functional scores and range of motion. One patient in Group A experienced donor site pain, while one patient in Group B showed significant graft resorption. Graft union was achieved in all patients. Two-thirds of patients in each group returned to their pre-injury level of sports participation.

Conclusion: Both techniques provided good clinical outcomes for anterior shoulder instability with glenoid bone loss. The modified Latarjet with iliac crest graft may offer slight advantages in functional outcomes and graft preservation but is associated with potential donor site morbidity. Larger, prospective studies are needed to definitively compare these techniques.

## Introduction

Anterior shoulder instability is a common orthopedic condition, affecting both athletic and non-athletic populations [1]. The Latarjet procedure, first described by Michel Latarjet in 1954, has become a widely accepted surgical technique for managing recurrent anterior shoulder instability, particularly in cases with significant glenoid bone loss [2,3].

The standard Latarjet procedure involves transferring the coracoid process along with its attached conjoint tendon (short head of biceps and coracobrachialis) to the anterior glenoid rim [4]. This technique provides both osseous and soft tissue restraints, creating what is often referred to as a "triple blocking" effect: increasing the glenoid arc, providing a sling effect from the conjoint tendon, and repairing the capsule to the coracoacromial ligament stump [5].

While the standard Latarjet procedure has shown good to excellent long-term outcomes in most patients, concerns remain regarding potential complications such as coracoid graft non-union, graft resorption, and persistent instability in cases of severe glenoid bone loss [6-8]. To address these issues, some surgeons have proposed modifications to the original technique, including the utilization of alternative graft sources [9].

The modified Latarjet procedure with bone graft, typically harvested from the iliac crest or other sites, aims to provide a larger osseous restoration of the glenoid while maintaining the dynamic stabilizing effect of the conjoint tendon [10]. Proponents of this modification argue that it may offer superior outcomes in cases of significant glenoid bone loss or revision scenarios [11].

However, the comparative efficacy of the modified Latarjet procedure with bone graft versus the standard Latarjet procedure remains unclear. This research aims to analyze and juxtapose the outcomes and steadiness provided by these two techniques in patients with repeated anterior shoulder dislocation.

## Materials and methods

The study was designed to be a retrospective comparative analysis of six subjects who opted for operative management for recurrent anterior shoulder dislocations. Subjects were segregated into two different groups: three subjects who underwent a modified Latarjet procedure with iliac crest bone graft (Group A) and three subjects who went through the standard Latarjet procedure utilizing the coracoid process (Group B). Operative procedures for both groups were completed under the same orthopedic consultant between January 2020 and December 2021. Inclusion criteria consisted of patients aged 18-45 years who were experiencing repeated anterior shoulder dislocations along with a large amount of glenoid bone loss (>20% of the glenoid width). Exclusion criteria included previous shoulder surgery, multidirectional instability, and concomitant rotator cuff tears.

Subjects went through a comprehensive pre-surgical assessment, which encompasses a meticulous review of their health record, clinical assessment, and imaging studies. Standard radiographs (anteroposterior, axillary, and scapular Y views) were acquired for the subjects. Additionally, computed tomography (CT) scans with 3D reconstruction were performed to accurately assess glenoid bone loss and plan the surgical procedure. Magnetic resonance imaging (MRI) was used to evaluate soft tissue structures. Preoperative functional assessments were conducted using the Constant-Murley score, the American Shoulder and Elbow Surgeons (ASES) score, and the Western Ontario Shoulder Instability Index (WOSI).

For Group A (modified Latarjet with iliac crest graft), the procedure began with harvesting a tricortical allograft from the iliac crest of the same affected side. The graft was shaped to match the glenoid defect. The usual deltopectoral approach was then utilized to access the glenohumeral joint. The subscapularis was split horizontally, and the anterior glenoid was uncovered. A prepared iliac crest graft had been affixed to the anterior glenoid using two cannulated screws of 3.5 mm each. The conjoint tendon was then sutured to the inferior capsule to provide additional dynamic stability.

For Group B (standard Latarjet), the coracoid process was separated from the base after detaching the pectoralis minor. The coracoid graft and conjoint tendon were relocated via a split within the subscapularis and affixed to the anterior glenoid using two 3.5 mm cannulated screws. The capsule had been repaired via the remnant of the coracoacromial ligament.

Subjects from both groups had identical standardized recovery plans. Operated limb immobilized in a sling for four weeks, during which assisted mobility exercises were started at two weeks. Active-assisted exercises began at four weeks, and strengthening exercises were introduced at eight weeks. Return to sports was permitted at six months, contingent upon satisfactory clinical and radiological outcomes.

The subjects were followed up at 14 days postoperatively, then 42 days postoperatively followed by regular follow-ups at three months, six months, and 12 months postoperatively. At each visit, a clinical examination was performed to assess range of motion, stability, and overall function. Radiographs were obtained at six weeks, three months, and 12 months for evaluating graft positioning and healing. CT scans were performed at six months to evaluate graft union and potential resorption.

Due to the small sample size, illustrative data was primarily utilized to summarize outcomes. Quantitative data was estimated by mean and standard deviation, while qualitative variables were delineated using frequencies and percentages. Due to a limited number of patients, formal statistical comparisons between the two groups were not performed.

## Results

The study compared two groups of three patients each, undergoing either a modified Latarjet procedure with iliac crest bone graft (Group A) or a standard Latarjet procedure using the coracoid process (Group B). Patient demographics and preoperative characteristics were similar between the groups, with comparable age ranges, glenoid bone loss, and preoperative functional scores (Table 1).

**Table 1:** Patient Demographics and Preoperative Characteristics

Characteristic	Group A (Bone Graft)	Group B (Coracoid)
Age (years)	28.3±4.7	30.7±5.2
Gender (M/F)	2/1	3/0
Dominant arm	2 (66.7%)	2 (66.7%)
Glenoid bone loss (%)	25.3±3.2	23.7±2.5
Preop Constant score	68.3±7.1	70.7±6.5
Preop ASES score	62.7±8.3	65.3±7.8
Preop WOSI score	1254.3±156.2	1198.7±143.9

At the 12-month follow-up, each set demonstrated a substantial improvement in shoulder stability and function (Table 2). Notably, no patients in either group experienced recurrent instability, suggesting that both procedures were effective in addressing the primary concern of shoulder instability. Functional outcomes, as measured by Constant, ASES, and WOSI scores, improved substantially in both groups. Group A showed slightly higher postoperative scores, but the small sample size precludes drawing definitive conclusions about superiority.

**Table 2:** Postoperative Outcomes at 12-Month Follow-Up

Outcome Measure	Group A (Bone Graft)	Group B (Coracoid)
Recurrent instability	0	0
Constant score	89.7±5.3	87.3±6.1
ASES score	91.3±4.7	88.7±5.5
WOSI score	384.7±72.3	421.3±85.6

Range of motion results were comparable between the two groups, with Group A demonstrating marginally better outcomes in forward flexion and external rotation (Table 3). This could potentially be attributed to the preservation of the coracoid process and its attached tendons in Group A, but again, the small sample size limits the ability to generalize these findings.

**Table 3:** Range of Motion at 12-Month Follow-Up

Motion	Group A (Bone Graft)	Group B (Coracoid)
Forward flexion (^0^)	168.3±7.6	165±8.7
External rotation (^0^)	62.7±5.5	58.3±6.1
Internal rotation (vertebral level)	T8±1	T9±1

In terms of complications, one patient in Group A experienced pain associated with iliac crest graft harvest, while no complications were reported in Group B (Table 4). This highlights a potential drawback of using an iliac crest bone graft. However, both groups achieved 100% graft union at six months. Interestingly, one patient in Group B showed significant graft resorption (>25%), which was not observed in Group A. This could suggest a potential advantage of using iliac crest graft in terms of graft preservation, but further investigation with larger sample sizes would be necessary to confirm this observation.

**Table 4:** Complications and Radiographic Outcomes

Outcome	Group A (Bone Graft)	Group B (Coracoid)
Complications	1 (33.3%), donor site pain	0
Reoperation	0	0
Graft union at 6 months	3 (100%)	3 (100%)
Graft resorption >25%	0	1 (33.3%)

Return to sports and activities was similar between the groups, with two-thirds of patients in each group restoring their level of activity to preinjury level (Table 5). This indicates that both procedures can effectively facilitate the return to sports and daily activities for most patients.

**Table 5:** Return to Sports/Activities at 12 Months

Outcome	Group A (Bone Graft)	Group B (Coracoid)
Full return to preinjury level	2 (66.7%)	2 (66.7%)
Partial return (lower level)	1 (33.3%)	1 (33.3%)
Unable to return	0	0

## Discussion

This small-scale comparative study aimed to evaluate the outcomes of a modified Latarjet procedure using an iliac crest bone graft versus the standard Latarjet procedure using the coracoid process. Despite the limited sample size, our findings suggest that both techniques can provide satisfactory clinical outcomes in terms of shoulder stability, function, and return to activities.

The absence of recurrent instability in both groups at 12-month follow-up aligns with the generally high success rates reported in the literature for Latarjet procedures. Mizuno et al. reported a recurrence rate of only 5.4% in a large series of 68 patients who underwent the classic Latarjet procedure, while Moroder et al found no recurrences in their series of 48 patients treated with iliac crest bone graft for glenoid reconstruction [12,13].

The improvement in functional scores observed in our study is consistent with previous research. Our study showed a trend toward slightly better functional outcomes in the iliac crest graft group, which warrants further investigation in larger cohorts.

Range of motion results in our study were comparable between groups, with a slight advantage in the iliac crest graft group. This contrasts with some previous studies, such as that by Ramhamadany and Modi, which found no significant difference in range of motion between patients treated with iliac crest bone graft and those treated with the classic Latarjet procedure [14].

The complication of pain from the graft site is a recognized issue with this technique. This highlights the need to carefully consider the risk-benefit ratio when choosing between these techniques.

Our observation of graft resorption in one patient from the coracoid group but none in the iliac crest graft group is interesting. Zhu et al. reported significant coracoid graft resorption in 90.5% of patients after the Latarjet procedure [8]. In contrast, Warner et al. found minimal resorption with iliac crest bone grafts [9]. Our results, albeit from a very small sample, seem to support these findings.

The similar rates of return to sports and activities between our two groups are encouraging and consistent with other studies. Bessière et al. reported that 83% of patients returned to sports after the Latarjet procedure, which is comparable to our findings [6].

While our analysis renders a meaningful insight, there are significant drawbacks, primarily due to a limited sample size. This precludes data assessment and limits the generalizability of our findings. Additionally, the short-term follow-up of 12 months may not capture long-term outcomes and complications.

## Conclusions

In conclusion, our preliminary results suggest that both the modified Latarjet procedure with iliac crest bone graft and the standard Latarjet procedure can provide good clinical outcomes for anterior shoulder instability with significant glenoid bone loss. According to our results, Group B showed significant bone resorption and less postoperative complication and would be the preferred method of surgical procedure. The choice between these techniques may depend on factors such as the extent of bone loss, surgeon experience, and patient characteristics. Larger, prospective studies with longer follow-ups are needed to definitively compare these techniques and guide clinical decision-making.
